# Long noncoding RNA *linc00598* regulates *CCND2* transcription and modulates the G1 checkpoint

**DOI:** 10.1038/srep32172

**Published:** 2016-08-30

**Authors:** Oh-Seok Jeong, Yun-Cheol Chae, Hyeonsoo Jung, Soon Cheol Park, Sung-Jin Cho, Hyun Kook, SangBeom Seo

**Affiliations:** 1 Department of Life Science, College of Natural Sciences, Chung-Ang University, Seoul 156–756, Republic of Korea; 2 Department of Biology, College of Natural Sciences, Chungbuk National University, Cheongju, Chungbuk 361-763, Republic of Korea; 3 Medical Research Center for Gene Regulation and Department of Pharmacology, Chonnam National University, Gwangju 501–746, Republic of Korea

## Abstract

Data derived from genomic and transcriptomic analyses have revealed that long noncoding RNAs (lncRNAs) have important roles in the transcriptional regulation of various genes. Recent studies have identified the mechanism underlying this function. To date, a variety of noncoding transcripts have been reported to function in conjunction with epigenetic regulator proteins. In this study, we investigated the function of *linc00598*, which is transcribed by a genomic sequence on chromosome 13, downstream of *FoxO1* and upstream of *COG6*. Microarray analysis showed that *linc00598* regulates the transcription of specific target genes, including those for cell cycle regulators. We discovered that *linc00598* regulates *CCND2* transcription through modulation of the transcriptional regulatory effect of FoxO1 on the *CCND2* promoter. Moreover, we observed that knockdown of *linc00598* induced G0/G1 cell cycle arrest and inhibited proliferation. These data indicate that *linc00598* plays an important role in cell cycle regulation and proliferation through its ability to regulate the transcription of *CCND2*.

Long noncoding RNAs (lncRNAs) are noncoding transcripts longer than ~200 bp, which are expressed in a more cell type-specific fashion than protein-coding genes^1^. Transcriptomic analyses have shown that lncRNAs are dominantly transcribed in the eukaryotic genome. During the last years, they have drawn considerable attention owing to their participation in various cellular processes, such as cell cycle regulation, and their role in human diseases^2^,^3^,^4^. An example of an extensively studied lncRNA is *lncRNA-p21*, which can suppress the transcription of genes involved in apoptosis and cell cycle through physical association with hnRNP-K, and can also inhibit the translation of β*-catenin* and *Jun B* mRNA^5^,^6^. Other lncRNAs, such as *HURC* and *PANDA*, also play a role in cell cycle and apoptosis through regulating the expression of cell cycle-related proteins^7^,^8^.

However, the functional roles of lncRNAs remain obscure. Even though lncRNAs have been revealed to function as transcriptional and posttranscriptional regulators of protein-coding genes, the mechanisms that underlie these epigenetic roles are not yet fully understood. Many well-known lncRNAs have been reported to regulate transcription of neighboring genes on the same chromosome^9^,^10^,^11^. However, lncRNAs can also act as transcriptional *trans*-regulatory elements, without affecting the transcriptional regulation of their neighboring genes^8^,^12^,^13^. Some nuclear lncRNAs have been shown to regulate gene expression by guiding specific proteins to target gene loci, or by acting as scaffolds for the recruitment of epigenetic modifying enzymes and the formation of chromatin remodeling complexes^12^,^14^,^15^. Motivated by these studies, we hypothesized that certain lncRNAs can regulate the expression of cell cycle-related genes by interacting with specific transcription factors through yet unidentified mechanisms.

The FoxO subfamily of transcription factors consists of functionally related proteins, including the mammalian FoxO1 (FKHR), FoxO3a (FKHRL1), FoxO4 (AFX), and FoxO6^16^,^17^. It has been reported that these transcription factors are involved in regulating a variety of biological processes, including metabolism, cell cycle, cell death, DNA repair, and oxidative stress response, via modulating a variety of target genes^17^,^18^,^19^,^20^. Recent studies have shown that FoxO family members can promote cell cycle arrest at the G1/S boundary both by upregulating cell cycle inhibitors, such as p21 and p27, and by downregulating positive cell cycle regulators, such as CCND1 and CCND2^21^,^22^,^23^.

In this study, we investigated the function of *linc00598*, also known as TTL (Twelve-thirteen Translocation Leukemia gene), which is located on chromosome 13, 74 kb downstream of FoxO1, by microarray expression analysis of *linc00598* stable-knockdown cell lines. Analysis revealed that *linc00598* knockdown affects the expression of 156 genes, 119 of which are downregulated. One of the downregulated genes was *CCND2*, which functions as a cell cycle regulator and is negatively regulated by FoxO1, whereas *linc00598* was found to be able to interact with FoxO1. Furthermore, knockdown of *linc00598* caused cell cycle arrest at the G0/G1 boundary, significantly reducing cell proliferation. Our results reveal a novel mechanism of transcriptional regulation of *CCND2* by lncRNA *linc00598* and FoxO1.

## Results

### Characteristics of long noncoding RNA *linc00598*

**Figure 1:**
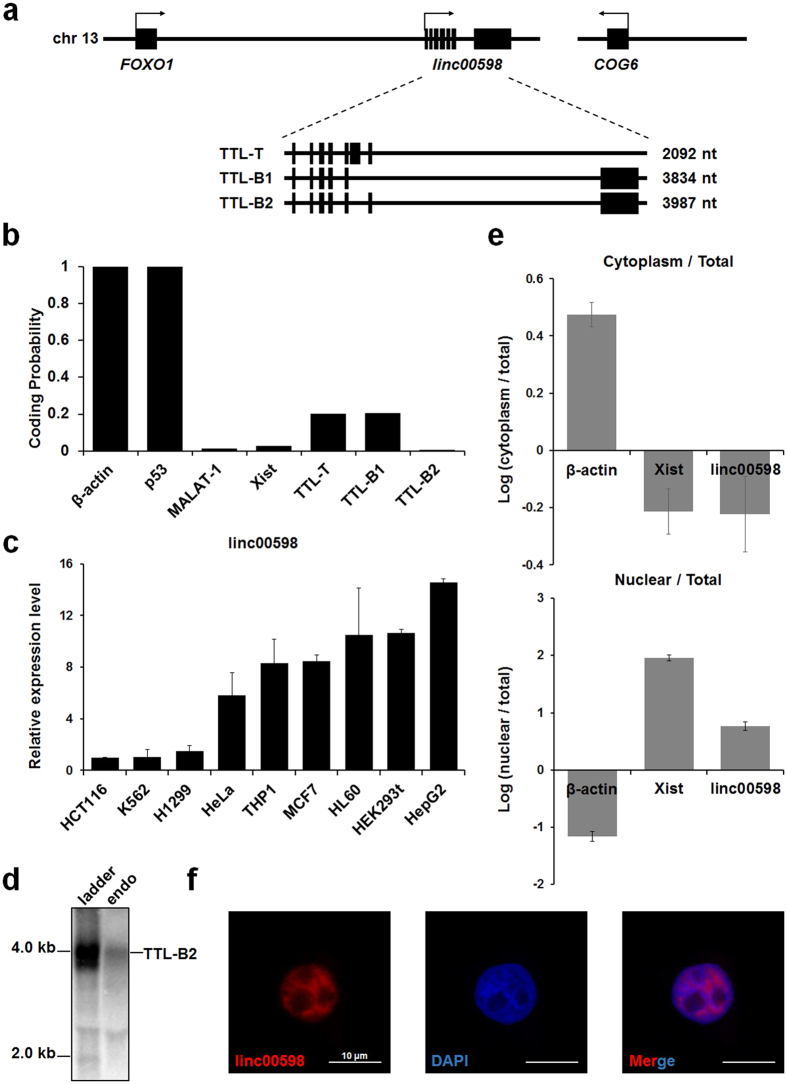
Expression of *linc00598*, a nuclear localized RNA, in human cells. (**a**) *linc00598*, which is transcribed by a sequence located between the *FoxO1* genomic locus and the *COG6* genomic locus, has three isoforms, *TTL-T*, *TTL-B1,* and *TTL-B2*. (**b**) Bioinformatic analysis of the coding potential of the three *linc00598* transcript variants. Results were obtained using the CPAT software. (**c**) The expression of *linc00598* was quantified by qRT-PCR in various human cell lines and normalized to *β-actin*. The HCT116 cell line was used as a calibrator, and its expression levels were arbitrarily set to “1.” (**d**) In order to determine the endogenous expression of *linc00598* isoforms, total RNA from HEK293t cells was analyzed by northern blot using random probes specific to the 5′ region of target transcripts. (**e**) Total RNA from HEK293t cells was separated into cytoplasmic and nuclear fractions and used to evaluate the expression levels of *linc00598* by qRT-PCR. *Xist* and *β-actin* RNA were quantified and used as references to calculate relative levels of each transcript and as controls to evaluate subcellular fractionation. The ratios of cytoplasmic to total, and nuclear to total RNA levels are shown. The results are expressed as mean ± S.D. (n = 3). (**f**) RNA-FISH was performed to detect *linc00598* (red) expression in HEK293t cells with Dig-labeled probes specific to the 5′ region of target transcripts. Images shown were acquired by laser scanning microscopy. Nuclei are colored blue due to DAPI. The white scale bar in all images represents 10 μm.

A previous study suggested that local changes in gene expression can be regulated by *cis*-acting lncRNAs, transcribed from sequences located in the same genomic region^24^. In our search for lncRNAs acting as *cis-*transcriptional regulators of FoxO1, we used the UCSC Genome Browser^25^ to examine the region close to the *FoxO1* genomic locus. We detected a long intergenic noncoding RNA, annotated as *linc00598*, located between *FoxO1* and *COG6* (Fig. 1a). To identify the coding potential of each variant of *linc00598*, we applied the coding potential assessment tool (CPAT) software^26^. All *linc00598* variants were found to produce noncoding transcripts similar to other lncRNAs, such as *MALAT-1* and *Xist* (Fig. 1b).

We performed qRT-PCR to determine the expression levels of *linc00598* in nine human cell lines. The colorectal cancer cell line HCT116, which displayed the lowest expression levels among the nine, was used as the calibrator, i.e., all other cell lines were compared to it to calculate the relative expression values that are depicted in Fig. 1c. The cell line with the highest levels of expression was HepG2. HEK293t cells also showed very high expression levels of *linc00598* and were chosen for further study.

To determine the isoforms of *linc00598* expressed in the HEK293t cell line, we performed northern blot using random probes specific to the 5′ region of the target transcripts. The results showed that *TTL-B2*, the longest isoform of *linc00598*, is the dominant, endogenously expressed isoform in HEK293t cells (Fig. 1d and Supplementary Fig. S1a,b).

A recent study supported that several nuclear localized lncRNAs play a role in transcription regulation^24^. To determine the localization of *linc00598*, we performed nuclear/cytoplasmic RNA fractionation in HEK293t cells (Fig. 1e). We observed that *linc00598* was mainly located in the nuclear compartment, similarly to *Xist*, a well-known nuclear lncRNA^27^. In order to confirm this result, we performed RNA fluorescence *in situ* hybridization (RNA-FISH) using an antisense *linc00598* RNA probe. As seen in Fig. 1f, *linc00598* in the HEK293t cells is exclusively retained in the nucleus. We conclude that *linc00598* is expressed in human cells and is localized in the nucleus.

### *linc00598* can regulate the transcription of genes related with cell cycle regulation

**Figure 2:**
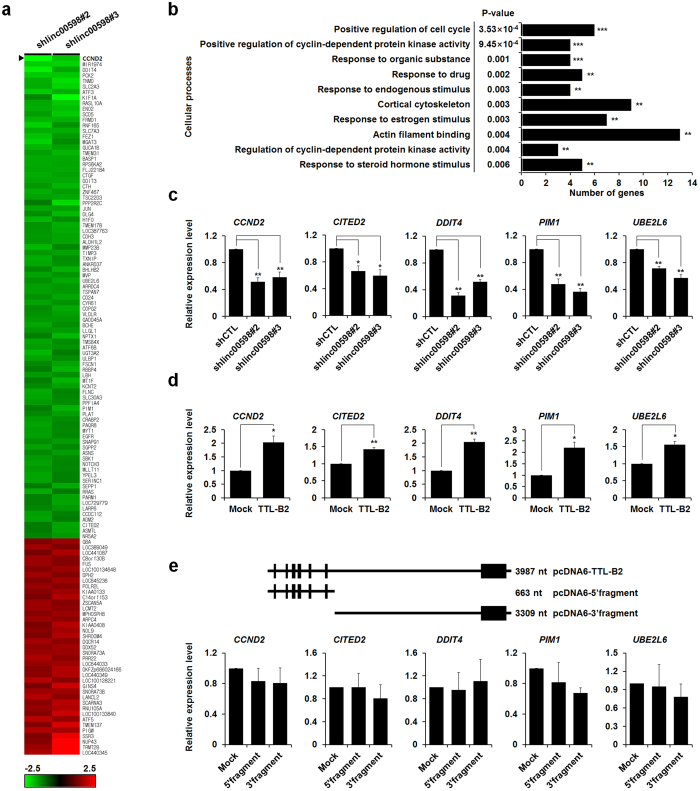
*linc00598* functions to regulate genes involved in cell cycle regulation. (**a**) Identification of *linc00598* target genes by hierarchical clustering; changes in the expression of a large number of genes in *linc00598* knockdown stable HEK293t cells are displayed. Upregulated and downregulated (by a factor of at least 1.4) gene clusters are represented by red and green, respectively. (**b**) Biological and molecular functional classification of *linc00598* target genes, using the annotation tool DAVID. **P < 0.01 and ***P < 0.001. (**c**–**e**) The mRNA levels of indicated genes in *linc00598* knockdown stable HEK293t cells and HEK293t cells transfected with indicated DNA constructs were analyzed by qRT-PCR and normalized to *β-actin*. The results are shown as means ± S.D. (n = 3). *P < 0.05 and **P < 0.01.

To determine the function of *linc00598* as a transcriptional regulator, we designed shRNAs targeting *linc00598* and produced stable *linc00598* knockdown HEK293t cells (Supplementary Fig. S2a). We performed microarray analysis using control shRNA and two shlinc00598 stable HEK293t cell lines (two replicates for each cell line) in order to identify *linc00598* target genes. As our aim was to filter out genes that did not display significant changes in expression, we chose only those genes whose expression values in the knockdown cells were higher or lower by a factor of at least 1.4 than that in the control cells. A total of 156 genes satisfied these criteria, of which 119 were downregulated and 37 upregulated (Fig. 2a). The fact that the vast majority (76%) of the differentially expressed genes were downregulated indicates that *linc00598* is mostly involved in target gene activation in HEK293t cells. However, the expression levels of *FoxO1* and *COG6*, which are located proximally to the *linc00598* genomic locus, did not exhibit changes in the *linc00598* knockdown cells. These results were confirmed by qRT-PCR (Supplementary Fig. S2b). Next, we performed functional annotation of the results by mapping these lists into the Gene Ontology (GO)^28^ and the Kyoto Encyclopedia of Genes and Genomes (KEGG) pathways^29^ databases, by utilizing the DAVID (Database for Annotation, Visualization and Integrated Discovery) software^30^,^31^. Results showed that a significant number of *linc00598*-regulated genes are involved in major biological processes, such as the regulation of cell cycle and regulation of cyclin-dependent protein kinase activity, as well as the modulation of responses to organic substances, drugs, and endogenous stimuli (Fig. 2b).

To confirm the changes in expression that were determined through the microarray analysis, we performed qRT-PCR for five *linc00598*-regulated genes, including those that exerted biological functions related to the cell cycle, in samples from two independent shlinc00598 stable HEK293t cell lines. All five genes displayed changes in expression consistent with the microarray data (Fig. 2c). The changes were further validated using ectopic transfection of the *linc00598* transcripts *TTL-B2* and *TTL-T* in HEK293t cells (Fig. 2d and Supplementary Fig. S2c, respectively).

To identify the part of the RNA sequence responsible for transcriptional regulation, we produced two different *linc00598* DNA constructs, containing either the 5′ (663 bp) or the 3′ region (3309 bp) of *TTL-B2*. Notably, overexpression of either DNA constructs did not influence the expression of the *linc00598* target genes (Fig. 2e), indicating that both parts of the RNA sequence are necessary for target gene regulation. Therefore, one of the *linc00598* isoforms, *TTL-B2*, appears to function as a transcriptional regulator of various target genes, in HEK293t cells.

### *linc00598* regulates transcription of *CCND2* through modulating accessibility of FoxO1 to the *CCND2* promoter

**Figure 3:**
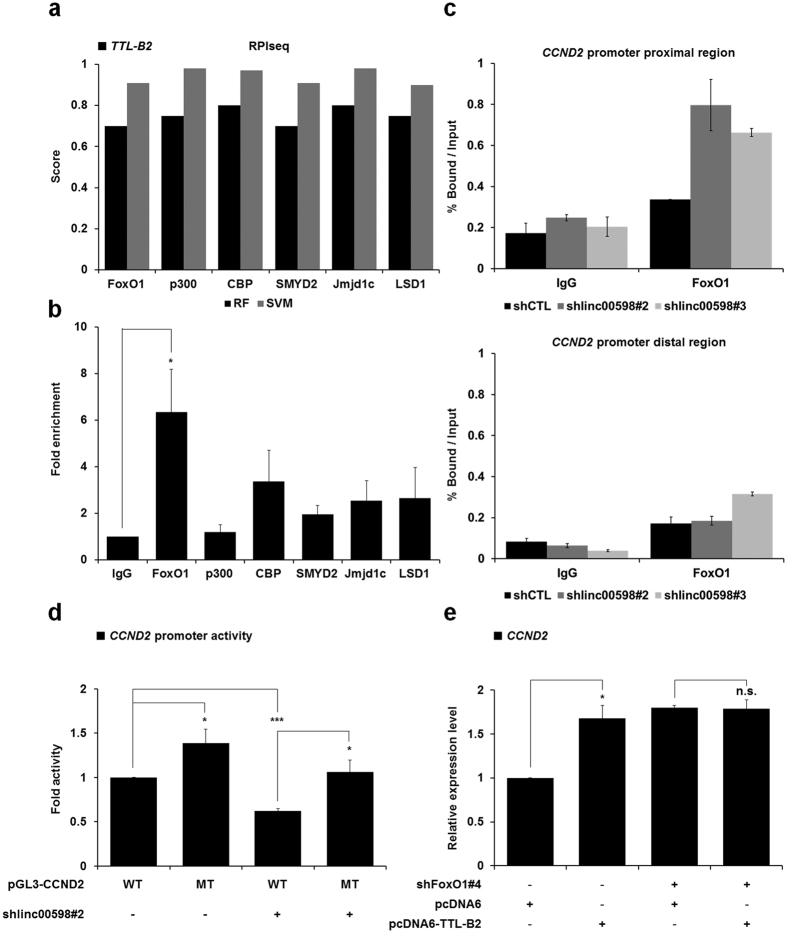
*linc00598* regulates the transcriptional activity of FoxO1 to the *CCND2* promoter. (**a**) Scores of the interaction probability between *TTL-B2* and various proteins as predicted by RPIseq. (**b**) *linc00598* interacts with FoxO1 in HEK293t cells. Total cell extracts from HEK293t cells were prepared and immunoprecipitated using indicated antibodies. Associated RNAs were purified and *linc00598* levels were measured using qRT-PCR. Results are expressed as fold enrichment relative to an isotype IgG control antibody. Values are means ± S.D. (n = 6). *P < 0.05. (**c**) ChIP analyses of the *CCND2* promoter region and a distal region in *linc00598* knockdown stable HEK293t cells were conducted using anti-IgG and anti-FoxO1 antibodies, and examined via qRT-PCR. The results are shown as mean ± S.D. (n = 3). (**d**) Control and shlinc00598#2 stable HEK293t cell lines were transfected with vectors containing the wild-type (pGL3-CCND2-WT) or the mutant (pGL3-CCND2-MT) *CCND2* promoter. Following transfection, cell extracts were assayed for luciferase activity. Luciferase activity was normalized to that of β-galactosidase. The results are expressed as means ± S.D. (n = 3). *P < 0.05 and ***P < 0.001. (**e**) FoxO1 knockdown stable HEK293t cells were transiently transfected with pcDNA-TTL-B2, analyzed by qRT-PCR and normalized to β-actin. The results are expressed as means ± S.D. (n = 3). *P < 0.05, **P < 0.01 and ***P < 0.001.

Among the differentially expressed genes identified, *CCND2* displayed the highest fold change (~2.17). Therefore, we tried to determine the mechanism of transcriptional regulation of *CCND2* by *linc00598*. It has been shown that *CCND2* is negatively regulated by FoxO1^23^, whereas our array data indicate that *linc00598* functions as a transcriptional activator for a variety of genes including *CCND2*. To test whether the protein levels of CCND2 are also regulated by FoxO1 and *linc00598*, we compared CCND2 levels in control shRNA, shFoxO1, and shlinc00598 cells. As expected, expression of CCND2 was negatively regulated by FoxO1 and positively regulated by *linc00598* (Supplementary Fig. S3a,b). We next analyzed the interaction between *linc00598* and transcriptional regulators related to *CCND2* expression by using RPIseq, a sequence-based predictive method with an accuracy ranging from 57–99% when faced with independent datasets of RNA-protein interactions^32^. We found that various transcriptional regulators, including FoxO1, p300, CBP, SMYD2, JMJD1C, and LSD1, had scores higher than 0.5, which indicates high interaction probabilities between *TTL-B2* and each of these proteins (Fig. 3a), and also suggests that these proteins have a high probability of participating in the *linc00598* transcriptional regulatory mechanism. To test whether *linc00598* indeed interacts with the aforementioned proteins, we performed RNA immunoprecipitation (RIP) assays using the indicated antibodies, followed by qRT-PCR. As seen in Fig. 3b, the results revealed that *linc00598* was associated with the FoxO1 protein, which, as mentioned above, is a negative transcriptional regulator of *CCND2*.

Accumulation evidence indicates that lncRNAs can change the transcriptional activity of specific target genes via interaction with proteins; for example, the ncRNA *Ctbp1* has been shown to increase the transcriptional activity of androgen receptors in prostate cancer cells^33^. To further investigate the mechanism of transcriptional regulation of *CCND2* by *linc00598* and FoxO1, we performed chromatin immunoprecipitation (ChIP) using anti-FoxO1 antibody in *linc00598* stable-knockdown cell lines, followed by qPCR assays. We observed that FoxO1 occupancy on the *CCND2* promoter increased when *linc00598* was knocked down, suggesting that *linc00598* is required for inhibition of FoxO1 accessibility to the promoter of *CCND2* (Fig. 3c).

Alternatively, the reduced presence of FoxO1 on the *CCND2* promoter could be a result of a reduction in total FoxO1 levels. To examine this possibility, we checked the expression of FoxO1 in shlinc00598 stable cells. The results showed that FoxO1 expression was not changed by depletion or overexpression of *linc00598* (Supplementary Fig. S3c). Moreover, as it has been reported that the transcriptional activity of FoxO1 is regulated by its localization^34^, we performed nuclear/cytoplasmic fractionation to determine whether the overexpression of *linc00598* changes the localization of FoxO1. As seen in Supplementary Fig. S3d, no such changes were observed.

To provide further evidence that the regulation of *CCND2* expression by *linc00598* is dependent on the modulation of the accessibility of FoxO1 to *CCND2* promoter, a reporter assay was performed using control and shlinc00598 stable HEK293t cells that were transfected with either wild type *CCND2* promoter, or a mutant form in which the FoxO1 consensus binding sequences had been altered. Consistent with our qRT-PCR and microarray data, *CCND2* promoter activity was decreased in the shlinc00598 stable cell line. Furthermore, *CCND2* promoter activity was rescued when we performed the luciferase assay using the shlinc00598 stable cell line that was transfected with the mutant promoter construct, suggesting that *linc00598* regulates *CCND2* by modulating FoxO1 binding on its promoter (Fig. 3d). To further demonstrate that *linc00598* regulates the transcription of *CCND2* by modulating the accessibility of FoxO1 to the promoter of *CCND2*, we performed qRT-PCR assays using control and shFoxO1 stable HEK293t cells transfected with *TTL-B2*. As expected, overexpression of *TTL-B2* in the control cells caused upregulation of *CCND2* expression. On the contrary, there was no significant change in *CCND2* expression in the shFoxO1 stable cell line (Fig. 3e), which is consistent with the results shown in Fig. 3d, which also suggest the existence of a FoxO1-mediated mechanism through which *linc00598* regulates *CCND2*. Taken together, these findings strongly suggest that *linc00598* regulates expression of *CCND2* through reducing the binding affinity of FoxO1 to the *CCND2* promoter.

### Knockdown of *linc00598* induces cell cycle arrest and inhibits cell proliferation

**Figure 4:**
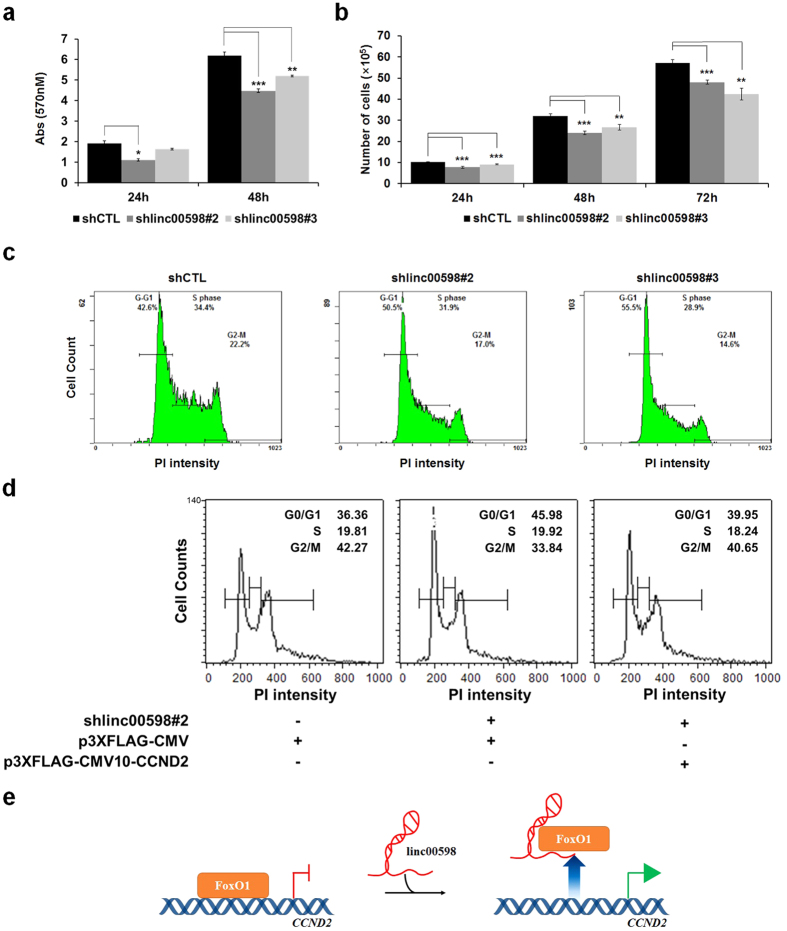
*linc00598* regulates cell proliferation via modulating cell cycle. (**a**) Cell proliferation was assessed through MTT assay in which *linc00598* knockdown stable HEK293t cells were used. Results are expressed as means ± S.D. (n = 3). *P < 0.05, **P < 0.01, and ***P < 0.001. (**b**) Cell counting assays were performed using *linc00598* knockdown stable HEK293t cells. The results are shown as means ± S.D. (n = 3). **P < 0.01 and ***P < 0.001. (**c**) Cell cycle phases of control and shlinc00598 stable HEK293t cell lines were analyzed by PI staining. Cells were fixed, stained with PI for 30 min, and analyzed by FACS. (**d**) Control and shlinc00598#2 stable cell lines were transfected with the indicated plasmids. Cell cycle phases of each cell line were assessed by PI staining. Cells were fixed, stained with PI for 30 min, and analyzed by FACS. (**e**) A model for regulation of *CCND2* transcription by *linc00598* through modulating accessibility of FoxO1 to the *CCND2* promoter.

We investigated the physiological consequences of *linc00598* knockdown in HEK293t cells. Since *linc00598* can regulate *CCND2*, which is a positive cell cycle regulator, we performed MTT assays to measure proliferation of control and shlinc00598 stable HEK293t cell lines. As shown in Fig. 4a, cell proliferation was reduced when *linc00598* was knocked down. Consistent results were obtained from cell counting assays, in which depletion of *linc00598* led to a decreased number of cells (Fig. 4b).

Since *linc00598* regulates expression of *CCND2*, we next examined whether *linc00598* knockdown affected the cell cycle in HEK293t cells. To this end, we performed propidium iodide (PI) staining followed by FACS analysis. As expected, the two *linc00598* knockdown cell lines exhibited an increase in G1 phase (about 8% and 13%, respectively) and a slight decrease in both S and G2-M phase (Fig. 4c). To determine whether the cell cycle arrest in the two shlinc00598 stable cell lines was caused by the depletion of CCND2, we performed another FACS analysis using the shlinc00598#2 stable cell line transfected with a CCND2 overexpression vector. Overexpression of CCND2 rescued the G1 arrest (G1 is reduced from 45.98% to 39.95%), suggesting that the cell cycle arrest caused by depletion of *linc00598* is caused by a decrease of CCND2 levels (Fig. 4d). These results are consistent with a previous study according to which, CCND2 contributes to the G1-S cell cycle transition^35^.

It has been reported that the CCND2/CDK4/p27 complex is required for nuclear translocation of CCND2^36^. Since the expression levels of CDK4 and p27 were not influenced in shlinc00598 stable HEK293t cell lines (Supplementary Fig. S4a,b), we examined the localization of CCND2 and CDK4 by performing nuclear/cytoplasmic fractionation. The amounts of CCND2 in both nucleus and cytosol were reduced in the two shlinc00598 stable HEK293t cell lines, whereas the mainly cytosolic localization of CDK4 was not affected by shlinc00598 knockdown (Supplementary Fig. S4c).

These results suggest that *linc00598* knockdown has no effect on the expression and localization of other complex components. Taken together, our results clearly indicate that *linc00598* affects cell proliferation through modulation of the G0-G1 checkpoint, via transcriptional regulation of *CCND2*.

## Discussion

Recent studies have revealed that various lncRNAs are transcribed in large amounts in the eukaryotic genome and these noncoding transcripts are involved in the regulation of gene expression and various biological processes including the cell cycle^3^,^4^. An example is *NcRNA*_*CCND1*_, also called pncRNA (promoter-associated non-coding RNA), that is transcribed from the 5′ regulatory region of *CCND1* and negatively regulates *CCND1* by recruiting the RNA binding protein, TLS (translocated in liposarcoma)^37^,^38^. *Gadd7* is another lncRNA involved in cell cycle control, specifically regulating *Cdk6* expression in a post-translational manner. *Gadd7* is transcriptionally induced by DNA damage caused by UV radiation and directly binds to TDP-43 (TAR DNA binding protein). This binding inhibits the interaction between TDP-43 and *Cdk6* mRNA, resulting in the degradation of *Cdk6* mRNA^39^.

*linc00598*, also known as the *TTL*, is located at the locus 13q14.11, downstream of the *FoxO1* genomic locus. It has three isoforms, *TTL-T*, *TTL-B1*, and *TTL-B2*. Notably, northern blot analysis showed that *TTL-B2* is the dominant expressed isoform in HEK293t cells. Results of RNA-FISH and qRT-PCR from fractionated RNA revealed a nuclear localization of *linc00598* and suggested that *linc00598* may probably serve as a *cis*- or *trans*-transcriptional regulator^24^. It has been reported that *CCAT1-L* (colorectal cancer specific lncRNA) localizes to its site of transcription and functions as a *cis*-transcriptional regulator of *MYC,* promoting long-range chromatin looping and interacting with the transcriptional regulator protein, CTCF^15^. Another example is *Paupar*, which interacts with chromatin at over 2,800 sites located on multiple chromosomes, and regulates target gene expression in *cis* and in *trans*. Our array data demonstrate that *linc00598* can regulate the expression of various target genes including cell cycle regulator *CCND2*, suggesting that the expression of *linc00598* could have an effect on cell cycle and proliferation. In order to determine the mechanism through which *CCND2* is regulated by *linc00598*, RIP and ChIP assays were performed. Results showed that *linc00598* can interact with FoxO1 and depletion of *linc00598* influence in accessibility of FoxO1 to the *CCND2* promoter. Furthermore, unlike the wild type, mutation of FoxO1 binding sites of the *CCND2* promoter rescued promoter’s activity in the shlinc00598 stable cell line. Consistently, ectopic expression of *linc00598* has no significant effect on the expression of *CCND2* in FoxO1-knockdown cells. These results indicate that *linc00598* modulates the accessibility of FoxO1 to the *CCND2* promoter. Finally, we demonstrated that *linc00598* can regulate cell cycle and cell proliferation by regulating the expression of *CCND2*, as *linc00598* knockdown reduces cell proliferation by downregulating *CCND2*.

Furthermore, a heatmap was generated from the hierarchical clustering of data from a systematic qRT-PCR analysis of a selected group of genes, including FoxO target genes (I), *linc00598* target genes (II), and Negative genes (III), in control shRNA, shlinc00598, siFoxO1, and shlinc00598/siFoxO1 cell lines. The heatmap revealed additional putative target genes under the control of the *linc00598*-FoxO1 regulatory mechanism, in ways similar to *CCND2* (Supplementary Fig. S5). Candidate target genes could be upregulated by *linc00598*, *such as CTGF* and *DDIT4*, or downregulated by *linc00598*, such as *FASLG*. These results indicate that part of the putative target genes of FoxO1 are regulated by *linc00598*, and suggest that the relationship between FoxO1 and *linc00598* needs to be examined further.

In this study, we provide for the first time information on the mechanism of transcriptional regulation of *CCND2* by *linc00598*. Specifically, we demonstrate that *linc00598* regulates the expression of *CCND2* through inhibiting the recruitment of FoxO1 to the *CCND2* promoter (Fig. 4e). The effects of this lncRNA on cell cycle regulation and cell proliferation indicate that *linc00598* has the potential to promote transformation in human cells. The exact mechanism through which *linc00598* affects the ability of FoxO1 to bind on the *CCND2* promoter is yet to be elucidated. Moreover, further studies are needed to determine the physiological significance of *linc00598*.

Taken together, the results of our study strongly indicate that *linc00598* regulates *CCND2* in *trans* through modulation of the accessibility of FoxO1 to the*CCND2* promoter.

## Methods

### Plasmid Constructs

To construct mammalian expression vectors, we employed modified pcDNA6-HA-myc-his plasmids (Invitrogen) to create expression vectors for *TTL-T*, *TTL-B2* (whole), the 5′ fragment (663 bp) of *TTL-B2*, and the 3′ fragment (3309 bp) of *TTL-B2*. The pOTB7-CCND2 (hMU010514) expression vector was obtained from the Korean Unigene Information (KUGI) collection. The *CCND2* coding sequence was subcloned into the mammalian expression vector p3XFLAG-CMV10 (Sigma). The *CCND2* promoter region (−463 to 0) was amplified from human genomic DNA and inserted into the *Kpn*I/*Hind*III sites of the pGL3-basic vector (Promega). The three FoxO1 binding elements (TATTT) of the cloned promoter were replaced with mutated elements (TGCCT or CGCCG) by site-directed mutagenesis. Short hairpin RNAs (shRNAs) against human *linc00598* and *FoxO1* were designed using the siRNA sequence designer software (Clontech). The double-stranded oligonucleotides used for shRNA plasmid construction were produced using primers from the 5′ to the 3′ end (Supplementary Table S1). The oligonucleotide for FoxO1 siRNA was introduced into the pBabe-dual vector using primers from the 5′ to the 3′ end (Supplementary Table S1). These oligonucleotides were inserted into the AgeI/EcoRI site of the pLKO.1 TRC vector.

### Antibodies

Antibodies against β-actin (sc-47778), CBP (sc-369), CDK4 (sc-260), FKHR (sc-374427), H3 (sc-8654), JMJD1C (sc-83420), LSD1 (sc-271720), p300 (sc-585), SMYD2 (sc-130879; Santa Cruz Biotechnology), Normal Mouse IgG (12-371; Millipore), β-tubulin (T4026; Sigma), and CCND2 (#3741; Cell Signaling) were employed.

### Cell Culture

HeLa and HEK293t cells were grown in Dulbecco’s modified Eagle’s medium (DMEM), whereas H1299, HCT116, HepG2, HL60, K562, MCF7, and THP1 cells were grown in RPMI-1640 containing 10% heat inactivated fetal bovine serum and 0.05% penicillin-streptomycin, at 37 °C in a 5% CO_2_ atmosphere. HEK293t cells were seeded in a 60 mm plate at a density of 4.0 × 10^5^ cells per well and transfected with the indicated constructs using polyethylenimine (Sigma). After 48 h of incubation, cells were harvested and used for each of the experiments.

### Reverse Transcription and Real-time PCR

Total RNA was isolated from HEK293t cells, using RNAiso Plus (TaKaRa). The synthesized cDNA was quantified and then used for analysis of mRNA expression. The PCR primers used are presented in Supplementary Table S1. Dissociation curves were created after each PCR run to ensure the amplification of a single product of the appropriate length. The mean threshold cycle (C_t_) and standard error values were calculated from individual C_t_ values obtained from triplicate reactions. The normalized mean C_t_ values (ΔC_t_) were calculated by subtracting the mean C_t_ of **β***-actin*. ΔΔC_T_ was calculated as the difference between the control ΔC_t_ and the values obtained for each sample. The n-fold change in gene expression, relative to an untreated control, was calculated as 2^−ΔΔCT^.

### Chromatin immunoprecipitation analysis

ChIP analysis was performed as described previously^40^. Briefly, control and shlinc00598 stable HEK293t cells were harvested and cross-linked with the addition of 1% formaldehyde in the medium for 10 min at 37 °C, followed by the addition of 125 mM glycine for 5 min at room temperature. The cells were then lysed in SDS lysis buffer, and the samples were sonicated and immunoprecipitated using the indicated antibodies. The immunoprecipitates were eluted and reverse cross-linked. The DNA fragments were then purified and PCR-amplified for quantification using the respective primer pairs (Supplementary Table S1). Dissociation curves were generated at the end of each PCR run to confirm the amplification of a single product of the expected length. The mean threshold cycle (C_t_) and standard error values were calculated from individual C_t_ values, obtained from duplicate reactions. The normalized mean C_t_ values (ΔC_t_) were calculated by subtracting the mean C_t_ of the input from that of the anti-CCND2 immunoprecipitated sample.

### MTT Assay

Control and shlinc00598 stable HEK293t cells were seeded in 48-well plates (8 × 10^4^ cells per well). After 24, 48, and 72 h, 3-(4,5-dimethylthiazol-2-yl)-2,5-diphenyltetrazolium bromide (MTT) was added to the cells at a final concentration of 0.5 mg/mL; after the addition, cells were further incubated for 4 h at 37 °C. The medium was then removed by aspiration, and DMSO was added (200 μl/well). OD values were determined on an ELISA reader (Biochrom) at a wavelength of 570 nm. The value of a blank sample, containing DMSO alone, was measured and subtracted from all values to correct for background in measurements.

### FACS Analysis

To assess the cell-cycle profile, control and shlinc00598 stable HEK293t cells were prepared. Control and shlinc00598 stable HEK293t cells transfected with the indicated plasmids were also seeded and harvested 48 h later. Cells were trypsinized, rinsed and fixed in ice-cold 70% ethanol for 30 min. Immediately before flow cytometric analysis, the cells were treated with RNase A (100 μg/mL) and stained with propidium iodide (PI, Sigma) for 30 min, then subjected to fluorescence-activated cell sorting (FACS) analysis using a BD Accuri C6 cytometer (BD Biosciences). Data were analyzed using BD Accuri C6 software (BD Biosciences).

### RNA Immunoprecipitation Analysis

We followed a modified version of the RIP protocol^41^. HEK293t cells were washed and then lysed with polysome lysis buffer (100 mM KCl, 5 mM MgCl_2_, 10 mM Hepes pH 7.0, 0.5% NP-40, 1 mM DDT, and 100 units/mL RNase out (Invitrogen), supplemented with a protease inhibitor cocktail. Cell extracts were then incubated with the indicated antibodies at 4 °C overnight. The next day, protein A/G-agarose beads (GenDEPOT) were added, and the samples were incubated at 4 °C for 4 h. Beads were then collected, washed five times with NT2 buffer, containing 50 mM Tris-HCl (pH 7.4), 150 mM NaCl, 1 mM MgCl_2_, and 0.05% NP-40). RNAiso Plus was added to the beads to extract protein-interacting RNA, which was then treated with RNase-free DNase I (TaKaRa) and measured by qRT-PCR assays.

### Microarray Analysis

For *linc00598* target gene profiling, we used the Illumina HumanHT-12 v4 Expression BeadChip (Illumina), which includes a bead pool of more than 47,231 unique bead types corresponding to 28,688 RefSeq annotated transcripts. Total RNA (0.55 μg) isolated from control and shlinc00598 stable HEK293t lines was reverse transcribed and amplified according to the protocols described in the Illumina TotalPrep RNA amplification kit manual (Ambion). *In vitro* transcription was then carried out to generate cRNA (0.75 μg), which was hybridized onto each array (two replicates for each condition) and then labeled with Amersham fluorolink streptavidin-Cy3 (GE Healthcare Bio-Sciences). The array was then scanned using the Illumina Bead Array Reader Confocal Scanner. Array data export processing and analysis were performed using Illumina GenomeStudio v2011.1 (Gene Expression Module v1.9.0). This data set was submitted to the Gene Expression Omnibus under submission number GSE80514. Array probes were transformed by logarithm and normalized by quantile method. Gene enrichment and functional annotation analysis for the significant probe list were performed using the DAVID software (http://david.abcc.ncifcrf.gov/home.jsp).

### Analysis of nuclear and cytoplasmic *linc00598* abundance

Nuclear and cytoplasmic RNA were isolated from untransfected cells for analysis of endogenous *linc00598* expression and localization. Cells growing in 100 mm dishes were rinsed twice with ice-cold 1 × PBS, harvested in 1 mL ice-cold 1 × PBS by scraping, and centrifuged at 1,000 rpm for 10 min. Cell pellets were resuspended by gentle pipetting in 200 μL lysis buffer A containing 10 mM Tris (pH 8.0), 140 mM NaCl, 1.5 mM MgCl_2_, and 0.5% NP-40, then incubated on ice for 5 min, and centrifuged at 1,000 × *g* for 3 min at 4 °C. The supernatant (cytoplasmic fraction), was added to 1 mL RNAiso Plus for RNA isolation and purification. Nuclear pellets underwent two additional rinses with lysis buffer A and a final washing step with lysis buffer A containing 1% Tween-20 and 0.5% deoxycholic acid. Purified nuclear pellets were then resuspended in 1 mL RNAiso Plus. Both RNA samples were treated with RNase-free DNase I, converted to cDNA, and quantified by qRT-PCR assays.

### RNA fluorescence *in situ* hybridization

HEK293t cells on PLL-coated cover glass were fixed in 1 × PBS with 4% paraformaldehyde for 15 min, then treated with 0.2 N HCl for 10 min, followed by incubation with 20 μg/mL proteinase K (Biofact) for 5 min at 37 °C. After undergoing acetylation in a solution containing 0.1 M triethanolamine (pH 8.0) and 0.1% acetic anhydride, the cells were then rinsed three times with 1 × PBS. Post fixation was performed using 4% paraformaldehyde for 20 min and rinsed three times with PBT (1 × PBS plus 0.1% Tween20). Prehybridization was carried out at 64 °C overnight in hybridization buffer (50% deionized formamide, 5 × SSC, 1 × Denhardt’s solution, 0.1% CHAPS, 100 μg/mL heparin, 0.1% Tween 20, and 100 μg/mL tRNA). The prehybridization buffer was replaced with fresh hybridization buffer containing 2 ng/mL of the *linc00598* probe and incubated at 64 °C overnight. After washing, samples were incubated at room temperature for 2 h in PBT containing 1% blocking reagent (Roche), then incubated at 4 °C for another 16 h with 1:2,000 Anti-DIG/POD antibody (Roche). After incubation, the color reaction was carried out using a tyramide kit (Tyramide Signal Amplification (TSA) Plus Cyanine 3/Fluorescein System, Perkin Elmer Lifer Sciences) at a 1/50 dilution, and leaving the samples in the dark for 10 min. After washing, samples were stained with 4′,6-diamidino-2-phenylindole (DAPI, Sigma) to visualize cell nuclei. Stained samples were rinsed in PBT overnight, then mounted in Fluoromount-G (SouthernBiotech) and examined by confocal laser scanning microscopy in a LSM700 microscope (Carl Zeiss Microscopy).

### Northern blot analysis

Total RNA samples prepared as described above were denatured at 65 °C for 10 min in a double volume of formamide-formaldehyde loading buffer, and then separated by electrophoresis in a 1.0% GTG agarose gel containing 1.85% formaldehyde. The gel was transferred to Hybond-XL nylon membranes (GE Healthcare) using 20 × standard saline citrate. The random hexamer probes used for *linc00598* detection were synthesized using a Random Primed DNA Labeling Kit (Roche), whereas the PCR products containing the 5′ region of target transcripts were used as a template. The sequences of the primers of the PCR reaction, linc00598-probe-F and linc00598-probe-R, are shown in Supplementary Table S1.

### Luciferase assay

Luciferase assays were conducted using a *CCND2* promoter reporter system, containing either wild type or a mutant form of the promoter. Control and shlinc00598 stable HEK293t cells were transfected with the indicated DNA constructs, using PEI. Cells were harvested after 48 h and assayed for luciferase activity, using a luciferase assay system (Promega). Each value is the mean of five replicates from a single assay. All experiments were performed at least three times.

### Statistical analysis

The results are expressed as means ± S.D of three or more independent experiments. Differences between groups were evaluated via Student’s *t*-tests, performed with Microsoft Office Excel. A *P* < 0.05 was considered statistically significant. The heatmap analysis was performed using the MeV v4.9 genomic analysis software (open source).

## Additional Information

**How to cite this article**: Jeong, O.-S. *et al.* Long noncoding RNA *linc00598* regulates *CCND2* transcription and modulates the G1 checkpoint. *Sci. Rep.* **6**, 32172; doi: 10.1038/srep32172 (2016).

## Supplementary Material

Supplementary Information
