# Supplementary material for: Long noncoding RNA linc00598 regulates CCND2 transcription and modulates the G1 checkpoint
Source: Sci Rep. 2016 Aug 30;6:32172. doi: 10.1038/srep32172 (PMC5004135; doi:10.1038/srep32172)
Supplement: Supplementary Information [file srep32172-s1.pdf]

Supplemental Information

Long noncoding RNA *linc00598* regulates *CCND2* transcription and modulates the G1 checkpoint

Oh-Seok Jeong[1], Yun-Cheol Chae[1], Hyeonsoo Jung[1], Soon Cheol Park[1], Sung-Jin Cho[2], Hyun Kook[3] & SangBeom Seo[1*]

[1]Department of Life Science, College of Natural Sciences, Chung-Ang University, Seoul 156-756, Republic of Korea

[2]Department of Biology, College of Natural Sciences, Chungbuk National University, Cheongju, Chungbuk 361-763, Republic of Korea

[3]Medical Research Center for Gene Regulation and Department of Pharmacology, Chonnam National University, Gwangju 501-746, Republic of Korea

***Correspondence: sangbs@cau.ac.kr**

Name	Sequence (5'- to -3')	Purpose
shlinc00598 #1 sense	CCGGGTGGCATGATCTCGGCTAACTCTCGAGAGTTAGCCGAGATCATGCCACTTTTTG	shRNA
shlinc00598 #1 anti-sense	AATTCAAAAAGTGGCATGATCTCGGCTAACTCTCGAGAGTTAGCCGAGATCATGCCAC	shRNA
shlinc00598 #2 sense	CCGGGATGATGGCTGTTGGCCGAATCTCGAGATTCGGCCAACAGCCATCATCTTTTTG	shRNA
shlinc00598 #2 anti-sense	AATTCAAAAAGATGATGGCTGTTGGCCGAATCTCGAGATTCGGCCAACAGCCATCATC	shRNA
shlinc00598 #3 sense	CCGGGCTGAAGGAACGAGTGATAACCTCGAGGTTATCACTCGTTCCTTCAGCTTTTTG	shRNA
shlinc00598 #3 anti-sense	AATTCAAAAAGCTGAAGGAACGAGTGATAACCTCGAGGTTATCACTCGTTCCTTCAGC	shRNA
shFoxO1 #4 sense	CCGGCAGGACAATAAGTCGAGTTATCTCGAGATAACTCGACTTATTGTCCTGTTTTTG	shRNA
shFoxO1 #4 anti-sense	AATTCAAAAACAGGACAATAAGTCGAGTTATCTCGAGATAACTCGACTTATTGTCCTG	shRNA
siFoxO1 #4 cloning sense	AAAGCAGGACAATAAGTCGAGTTAT	siRNA cloning
siFoxO1 #4 cloning anti-sense	AAAAATAACTCGACTTATTGTCCTG	siRNA cloning
CCND2 promoter F	GAGTGAGGCGCGAAACCG	ChIP
CCND2 promoter R	TGAGGGATTAGGTCCGGCTC	ChIP
CCND2 distal F	GGTGAAAAGCAAGGTACCCG	ChIP
CCND2 distal R	TGTTTCACAAATGAATTCTGCCCT	ChIP
linc00598-probe-F	CGCTGTAGAGCTCCGATGAT	Random probe
linc00598-probe-R	CAGAAGCTGAGGCACATTCC	Random probe
linc00598 F	TACAGCAAACAATGGACTATTGACTAA	qRT-PCR
linc00598 R	ACTAGGATCTTAAGGCAGAGAAGTGAG	qRT-PCR
XIST F	GCAGGTCCAAGAAATTTGAACAC	qRT-PCR
XIST R	AGAGTGCCAGGCATGTTGATC	qRT-PCR
CCND2 F	CTACTGGGTCATCCTTGGTC	qRT-PCR
CCND2 R	TAACCTTTCAGTCCCAGAGC	qRT-PCR
CITED2 F	CCACTACATGCCGGATTTGC	qRT-PCR
CITED2 R	TCTCGGAAGTGCTGGTTTGTC	qRT-PCR
DDIT4 F	AAGGGACCAAGTGTGTTTG	qRT-PCR
DDIT4 R	GCTCAACTCTGCAGTACAC	qRT-PCR
PIM1 F	GCTGTGCTGGGAGAAATACT	qRT-PCR
PIM1 R	GGTCTTGGCTTTGAAACAGT	qRT-PCR
UBE2L6 F	TCATGTTCTGACCCTCTGTG	qRT-PCR
UBE2L6 R	AACCTGGGAGTGAGTCCATA	qRT-PCR
FOXO1 F	TACGAGTGGATGGTCAAGAG	qRT-PCR
FOXO1 R	ATGAACTTGCTGTGTAGGGAC	qRT-PCR
COG6 F	CCACCACCTGATCTTGGACC	qRT-PCR
COG6 R	GCTGTGCCTAAATTGCTGGC	qRT-PCR
p27 F	GTTTGGAGAGCGGCTGGGTT	qRT-PCR
p27 R	CAAGCGGAGAGGGTGGCAAA	qRT-PCR
linc00598(exon5,7)-F	TCAGAAAATCAGGCCTCCTC	RT-PCR
linc00598(exon5,7)-R	CAGACTGGCCAACAGAGACA	RT-PCR

Table S1. Primer pairs used in this study

Table S1

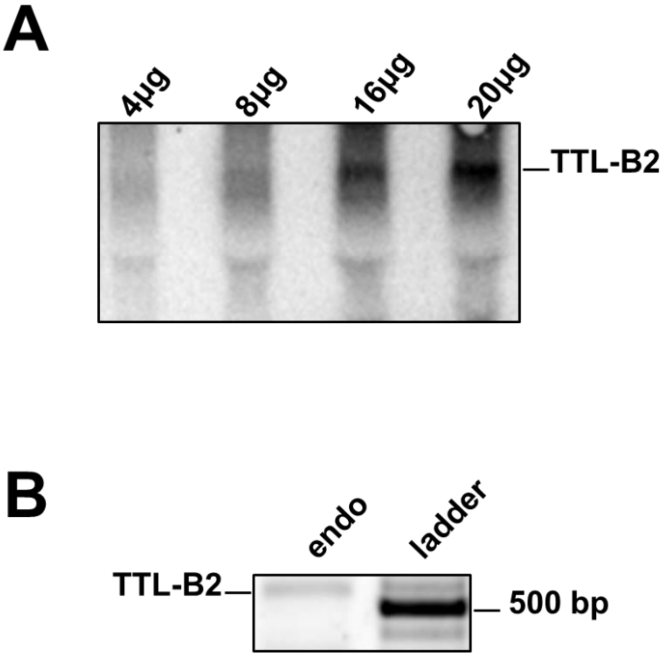

Figure S1. The *TTL-B2* isoform of *linc00598 is* dominantly expressed in HEK293t cells.

(a) Northern blot analysis using various amounts of total RNA of HEK293t cells was performed.

(b) RT-PCR between the TTL-B2 exons 5 and 7 amplified only one size variant in HEK293t cell (TTL-B1 ; 410 bp, TTL-B2 ; 550 bp).

Figure S1

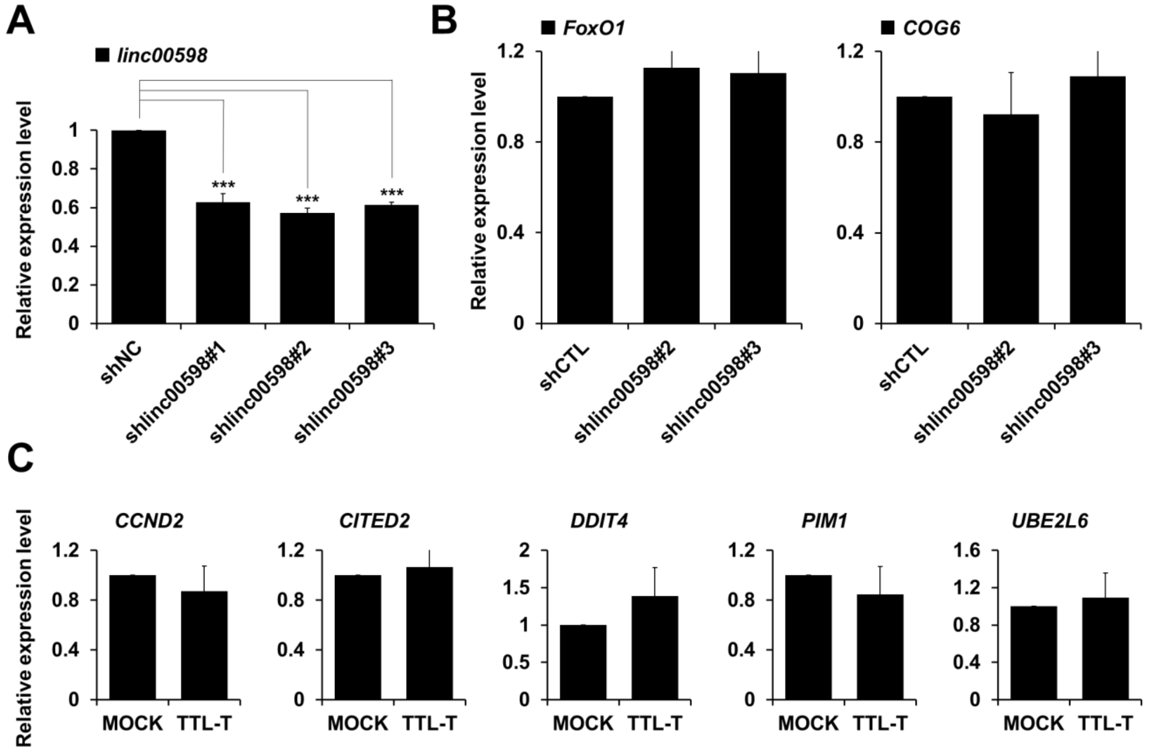

Figure S2. ***TTL-B2*****, one of the** ***linc00598*** **isoforms, is not a** ***cis*****-acting transcriptional regulator.**

(a) Expression levels of *linc00598* in control and three shlinc00598 stable HEK293t cell lines were analyzed by qRT-PCR and normalized to *β-actin*. The results are shown as means ± S.D. (n = 6), $***P < 0.001$. (b) The mRNA levels of *FoxO1* and *COG6* in control and shlinc00598 stable HEK293t cell lines were analyzed by qRT-PCR and normalized to *β-actin*. The results are shown as means ± S.D.(n = 3). (c) The mRNA levels of indicated genes in HEK293t cells transfected with TTL-T were analyzed by qRT-PCR and normalized to *β-actin*. The results are expressed as means ± S.D. (n = 3).

Figure S2

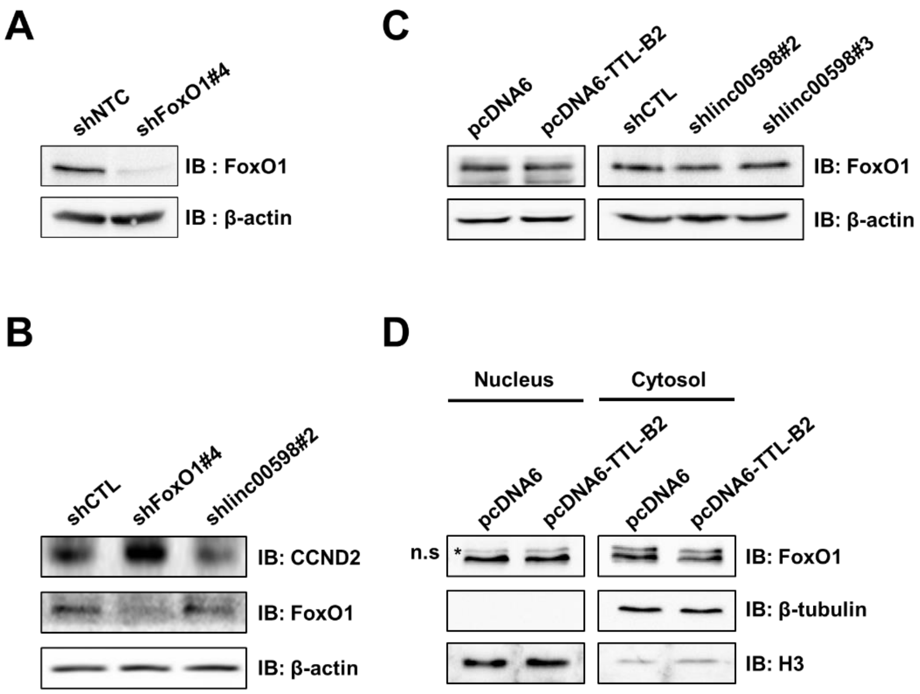

Figure S3. *Linc00598* regulates the expression of CCND2 without affecting the expression or the localization of FoxO1

(a) FoxO1 levels were measured in control and shFoxO1 stable HEK293t cells by Western blot analysis. β-Actin was used as a loading control. (b) Expression levels of CCND2 and FoxO1 were measured in control shRNA, *FoxO1* knockdown, and *linc00598* knockdown stable HEK293t cells by Western blot analysis. β-Actin was used as a loading control. (c) FoxO1 was detected by Western blot analysis in *linc00598* knockdown stable HEK293t cells and HEK293t cells transfected with pcDNA6-TTL-B2. β-Actin was used as a loading control. (d) Nuclear and cytoplasmic FoxO1 levels were measured by Western blot analysis in HEK293t cells transfected with pcDNA6-TTL-B2 after nuclear/cytoplasmic fractionation. Histone H3 and β-tubulin were used as loading controls for the nuclear and the cytoplasmic fraction, respectively.

Figure S3

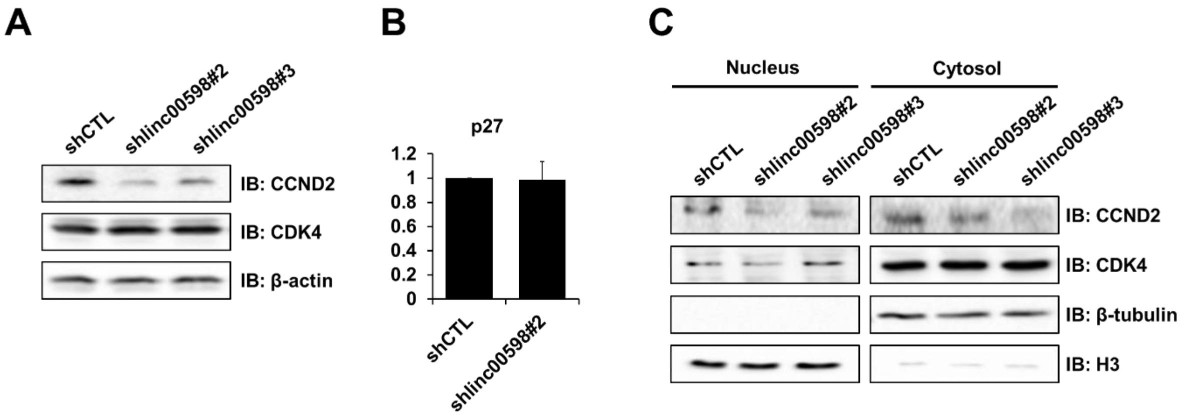

Figure S4. Nuclear translocation of CCND2 was not affected by expression of *linc00598*. (a) CCND2 and CKD4 levels were quantified in control and shlinc00598 stable HEK293t cell lines by Western blot analysis. β-Actin was used as a loading control. (b) The mRNA levels of *p27* were measured by qRT-PCR in control and shlinc00598#2 stable HEK293t cell lines and normalized to *β-actin*. The results are expressed as means ± S.D. (n = 4). (c) Nuclear/cytoplasmic fractionation was performed in control and shlinc00598 stable HEK293t cell lines. CDK4 and CCND2 were detected by Western blot analysis. Histone H3 and β-tubulin were used as loading controls.

Figure S4

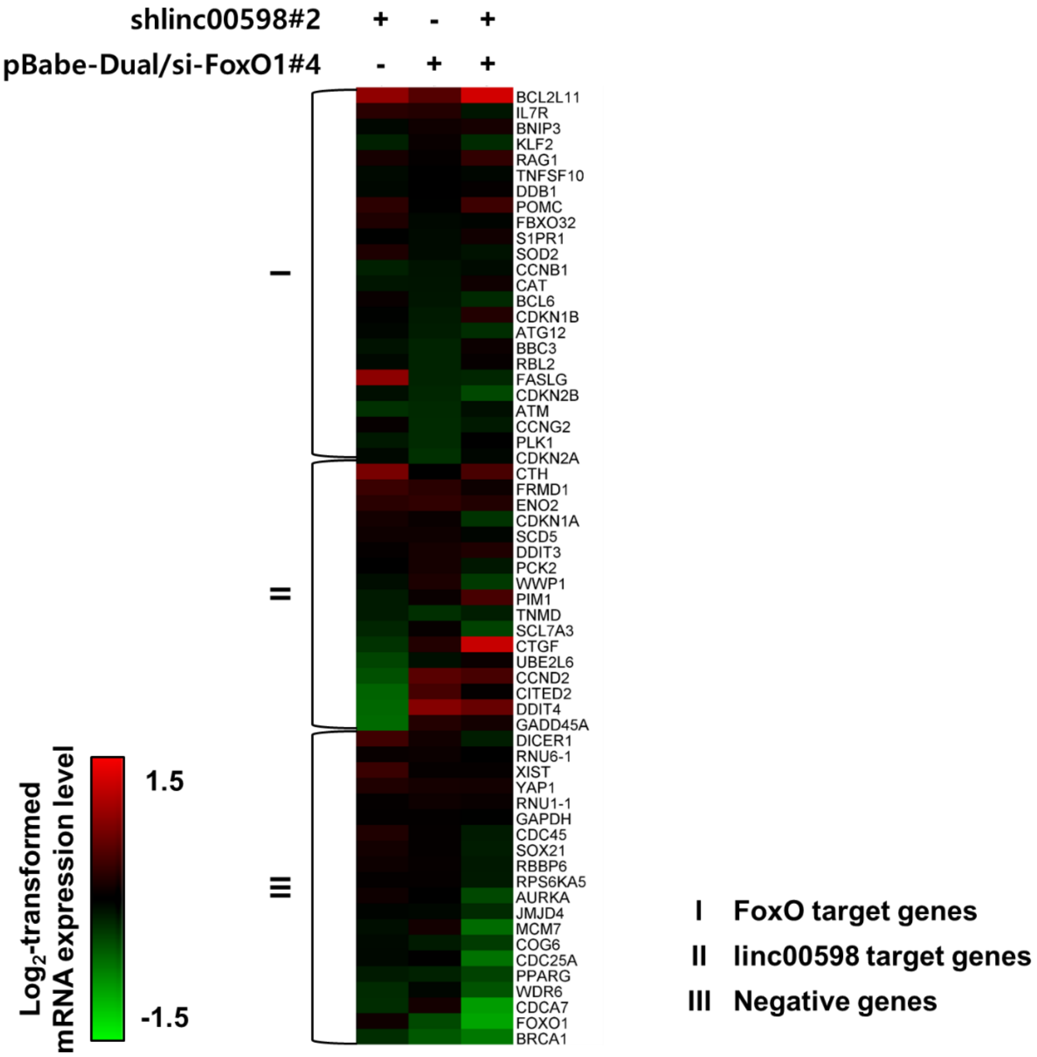

Figure S5. Systematic qRT-PCR analysis in control shRNA, shlinc00598, siFoxO1, and shlinc00598/siFoxO1 cells.

Control and shlinc00598 stable cell lines were transfected with the indicated plasmid. The mRNA levels of indicated genes in each sample were analyzed by qRT-PCR and normalized to *β-actin*. The qRT-PCR results were presented in a heat map using MeV v4.9 and the indicated genes were separated into three groups – FoxO target genes (I), *linc00598* target genes (II), and Negative genes (III). Genes with *P*-values of 0.1 or less were selected.

Figure S5
